# S14 Physical activity promotion in healthcare settings: Designing and implementing physical activity referral schemes

**DOI:** 10.1093/eurpub/ckae114.259

**Published:** 2024-09-26

**Authors:** Wolfgang Geidl, Mark Stoutenberg

**Affiliations:** Friedrich-Alexander University Erlangen-Nürnberg, Department of Sport Science and Sport; Durham University

## Abstract

**Purpose:**

This symposium will share findings, expertise and experiences from different countries on the design and implementation of physical activity referral schemes (PARS).

**Description:**

Physical activity promotion by healthcare professionals is a key strategy to increase population physical activity levels. This strategy has been outlined in the World Health Organisation’s (WHO) ‘Global Action Plan on Physical Activity’ (GAPPA) and the ‘Physical activity strategy for the WHO European Region 2016-2025‘. PARS are a promising intervention that allow healthcare professionals to advocate for physical activity and integrate its promotion into routine clinical care.

PARS are a heterogeneous group of interventions. Simpler PARS, for example, may consist only of a written description, while more complex PARS might include different healthcare professionals working together systematically, applying several behaviour change techniques in combination with physical activity practice, to connect patients to community resources. There is currently no consensus on the optimal PARS approach and implementation strategies. Implementers need specific knowledge, skills and methods; however, implementation is also influenced by structural and context-related factors in the healthcare system. Accordingly, a successful implementation of PARS needs co-productive capacity building within healthcare systems and proper support for healthcare professionals.

The symposium includes four contributions (3 research-oriented and 1 practise-oriented) related to the design and implementation of PARS in different countries.

1) EUPAP Feasibility Study. Practice transfer of a HEPA prescription model to other nine EU countries. From theory to practice. Sebastià Mas-Alòs

2) The association of physical activity referral scheme’ components with physical activity level, scheme uptake and adherence rates: a systematic review, meta-analysis with meta-regression. Wolfgang Geidl

3) The adoption and implementation of a physical activity referral pathway integrated into a major U.S. health system. Mark Stoutenberg

4) Designing a physical activity pathway in healthcare model for Irish Health Services. Sarah O’Brien

This symposium will provide guidance on the development of PARS and future successful implementation in other healthcare settings.

